# Menstrual health management and schooling experience amongst female learners in Gauteng, South Africa: a mixed method study

**DOI:** 10.1186/s12978-020-0896-1

**Published:** 2020-04-15

**Authors:** Tamaryn L. Crankshaw, Michael Strauss, Bongiwe Gumede

**Affiliations:** 1 Health Economics and HIV and AIDS Research Division (HEARD), University of KwaZulu-Natal, Westville Campus, Private Bag X54001, Durban, 4000 South Africa; 2 Legal Resources Centre, P.O. Box 9495, Johannesburg, 2000 South Africa

**Keywords:** Menstrual health management, MHM, Menarche, School absenteeism, Sexuality education, Sexual and reproductive health, Gender, South Africa

## Abstract

**Background:**

There has been increased attention to the menstrual health management (MHM) needs of girls and young women in Eastern and Southern Africa, relating to dignity, and to the potential link between the lack of access to sanitary products and school absenteeism. In the South Africa, there is inadequate evidence to guide appropriate national responses. This study explored the extent of access to modern sanitary products amongst female high school learners and the range of needs and challenges that they face in managing their menses in school settings in Gauteng, South Africa.

**Methods:**

We collected mixed method data from 10 schools in Sedibeng district between June and August 2018. The qualitative component consisted of in-depth interviews with female learners (*n* = 30), educators (*n* = 8) and mothers of female learners (*n* = 9) and focus group discussions (FGDs) with male learners (*n* = 7) and female learners (*n* = 10). Five hundred and five female learners were recruited into the quantitative component consisting of a self-administered survey focussing on factors associated with access to sanitary products.

**Results:**

The median age of survey participants was 17 years (interquartile range 16–18 years) and average age at menarche was 13.36 years. One in seven female learners reported not having enough sanitary products for every period in the last 3 months and this was reflected across the school quintiles. There was a complex interaction between menstrual-related challenges (physical discomfort, teasing, and feeling distracted in class) experienced by female learners, often amplified or compounded by factors in the school environment (unhygienic sanitation facilities and inadequate rest areas), and schooling participation and attendance. Girls who did not have enough products for every period in the last 3 months more likely reported missing school than those who reported sufficient products (46.27% vs 22.49% respectively, *p* < 0.001). However, there was no statistically significant difference between the groups in number of days missed.

**Conclusions:**

Provision of sanitary products is important but only one component of a comprehensive MHM response. Ongoing attention over the link between product access and absenteeism risks overlooking complex systemic and structural factors which can negatively impact the sexual and reproductive health of learners in the school context, and more broadly.

## Plain English summary

There has recently been a call for countries to provide female school learners with modern sanitary products because there is concern that girls who don’t have enough products are missing school. This can compromise their educational performance. However, not enough is understood around the challenges to access. This paper presents the findings from a study exploring the levels of access to modern sanitary products amongst female learners and the range of needs and challenges that they face in managing their periods in the school setting in Gauteng, South Africa. We collected data in 10 schools in Sedibeng district between June and August 2018. We conducted interviews with female learners, educators and mothers as well as group discussions with male and female learners. We also conducted a survey amongst female learners to try understand what factors played a role in creating challenges to access to products. We found that: 1) one in seven girls did not always have access to sanitary products highlighting the problem of unequal access; 2) there is a complex relationship between menstrual-related challenges, the school environment and classroom participation and school attendance; 3) menstrual health information needs were only one part of a number of other unmet sexual and reproductive health needs. We conclude that an exclusive focus on the link between lack of products and school absenteeism ignores the important role that the schooling environment plays in shaping girls’ experiences and the challenges they face during their periods.

## Background

The right to dignity and the right to education cannot be compromised by the circumstance of being born young, female and poor. These two fundamental principles underpin the growing attention to the ‘menstrual health management’ (MHM) needs of girls and women – a term which consciously locates a biological process within a human rights framework in which menstruation is inextricably linked with girls’ and young women’s empowerment, dignity, gender equality, and sexual and reproductive health (SRH) and well-being [1, 2]. It is in this context that momentum has been building to support the MHM needs of girls and women across the Eastern and Southern African (ESA) region, and which has found champions at all levels of society, from country leaders to grassroots organisations [2, 3]. The increasing number of ESA countries that have started providing free sanitary pads to girls in schools and/or have removed tax duties from sanitary products to enable better access bears testimony to the effectiveness of these advocacy efforts.

There are two dominant positions underlying the groundswell support for ensuring adequate MHM amongst girls and young women [4, 5]: the one relates to the issue of dignity and social justice; and the second to the possible link between poor MHM – in particular a lack of access to sanitary products - and school absenteeism amongst school-going girls and young women [2, 5–7]. While the first position resonates with the human rights regime underpinning the Sustainable Development Goals (SDGs), there remain large gaps in evidence supporting the latter position [5–8]. Increasingly, a more holistic approach to MHM is being called for by the scientific community based on accumulating evidence suggesting the need for a cross-sectoral response embedded in a broader sexual and reproductive health and rights framework [9].

Data emerging from the African context suggest that girls may miss school due to their menses [10–13]. However, there exist a number of reasons why a learner may miss school while menstruating, including physical symptoms, shame, secrecy and fear of leakage, access to products and/or ability to manage menstruation at school [5, 14–16]. Apart from the possible impact on a girls’ academic progression, challenges experienced by girls in managing their menses can also work to reinforce gender inequalities and further marginalize girls in low-income contexts [17]. While there are an increasing number of observational studies investigating the relationship between menstrual hygiene management and participation in school, a recent systematic review has highlighted the dearth of rigorous and comparable data to adequately determine the relationship between menstruation, access to products, schooling, health and psychosocial outcomes [18].

In South Africa, there has been significant mobilization by the civil society sector around the lack of access to sanitary products and school absenteeism amongst female learners. There have also been efforts at national level with the drafting of a Sanitary Dignity Policy Framework in 2017 to help set out the norms and standards for how the nine provinces within South Africa should address sanitary dignity. Although hampered by governance issues, 2018 saw some individual provinces taking the lead in attempting to establish provincially-based school distribution programmes for the provision of disposable sanitary pads to female learners. While the principle behind these efforts is commendable, the reality is that very little research has, to date, been undertaken in the South African context to help guide these efforts. Where local research exists, in the form of small qualitative studies, MHM challenges have been linked to poor conditions of water and sanitation facilities, lack of privacy and discreet waste disposal options, and outdoor sanitation facilities that are perceived as unsafe [19–21]. Ahead of a larger quantitative survey on the issue in South Africa, we conducted an exploratory study in one district of Gauteng province, South Africa to better ascertain the prevalence of access to modern sanitary products amongst female learners and to better understand the range of needs and challenges that female learners face in managing their menses in the school setting.

## Methods

### Overview

This was a mixed method study consisting of a qualitative and quantitative component. The qualitative component was exploratory in nature to determine the range of challenges from the perspective of female and male learners as well as educators and mothers. The primary objective for the quantitative component was to explore the extent of the challenge of access and any associated factors related to access.

### Study setting and sample

The qualitative component consisted of semi-structured individual interviews with 30 female learners, 8 educators and 9 mothers of female learners in the study schools. Additionally, we conducted 7 focus group discussions (FGDs) amongst male learners and 10 FGDs amongst female learners attending the study schools. We recruited 505 female learners attending the study schools into the quantitative component which consisted of a self-administrated survey. The main consideration for the sample size estimation is that the study is characterized as a pilot study and seeks to calculate the prevalence of access to modern menstrual hygiene products in two rural and eight urban schools. The initial sample size that was estimated was calculated as 235 young girls, assuming a total population in the schools of 5000 young girls across all 10 schools, 20% of whom would have regular access to modern menstrual hygiene products. This was calculated with a 5% precision and 95% confidence level. In order to calculate the prevalence in urban and rural schools separately, this sample size of 235 was multiplied by two, to get to a minimum sample size of 470. Allowing for an additional 6% for non-response the final sample size of 500 girls was used in this study. Fieldwork challenges and financial constraints to this small pilot study meant that it was not possible to achieve the full sample size for the rural schools. The final sample included between 44 and 50 female learners were sampled from each school for the survey. Table 1 shows the number of respondents recruited per component of the research. Data were collected between June 2018 and August 2018.

**Table 1:** Research sample recruited per school

School profile			Semi structured interviews			Focus Group Discussions		Survey
School ID	Learners per school^a^	Educators per school^a^	Educators	Mothers	Female learners	Male learners	Female learners	Female learners
A	1238	39	1	1	3	1	1	51
B	1110	30	1	1	3	1	1	50
C	403	18	1	1	3	1	1	50
D	951	33	1	1	3	1	1	52
E	1289	43	0	1	3	0	1	49
F	759	33	1	0	3	0	1	51
G	1463	41	0	1	3	0	1	53
H	1082	37	1	1	3	1	1	50
I	1261	43	1	1	3	1	1	50
J	613	18	1	1	3	1	1	49
		Total	8	9	30	7	10	505

^a^Figures come from the most recent EMIS data available at: https://www.education.gov.za/Programmes/EMIS/EMISDownloads.aspx

The study was conducted in 10 public schools in the Sedibeng district. Sedibeng is the second largest district in the Gauteng Province in South Africa but the second least densely populated. Sedibeng has the highest unemployment rate of any of the districts (39.3% compared to a provincial average of 28% in 2015) and although the district is highly urbanized (an estimated 93% in 2014), with most people living in towns and townships, poverty remains high (an estimated poverty rate of 21% in 2014) [22]. Sedibeng has a low density of high schools compared to the rest of the province, but this can in part be related to the fact that the population density is low. Ten of the 62 public schools in the Sedibeng district were purposively selected for inclusion in this study based on an existing working relationship between the schools and the research team. Two of the schools were classified as rural and eight as urban. Two schools from quintile 2, four schools from quintile 3 and four from quintile 4 were included in the study. They represent 16% of the public schools within the Sedibeng district (*N* = 62). Table 2 shows the number of urban and rural public schools, and number of public schools by quintile in the Sedibeng District versus those included in the study sample. Overall, this sampling strategy oversampled schools from Quintiles 3 and 4, and is not representative of schools in Quintiles 1 or 5. The sample is fairly representative of the urban/rural split in the district as shown in Table 2.

**Table 2:** Number of public schools by geographical area and quintile^a^ in the Sedibeng District vs number included the study sample

	Sedibeng Public Schools (Population)	Study schools (Sample)
Urban (%)	*N* = 52 (83.9%)	*n* = 8 (80%)
Rural (%)	*N* = 10 (16.1%)	*n* = 2 (20%)
Quintile 1 (%)	*N* = 8 (9.7%)	*n* = 0 (0%)
Quintile 2 (%)	*N* = 14 (22.9%)	*n* = 2 (20%)
Quintile 3 (%)	*N* = 14 (22.6%)	*n* = 4 (40%)
Quintile 4 (%)	*N* = 12 (19.4%)	*n* = 4 (40%)
Quintile 5 (%)	*N* = 16 (25.8%)	*n* = 0 (0%)
Grand Total	**N = 62 (100%)**	***n*** **= 10 (100%)**

^a^Public schools in South Africa are categorized by quintiles which determine how much government funding each school receives. Schools in the lower quintiles [1] are fees exempt schools in that they do not charge school fees while schools in quintile 4 and 5 receive limited government funding and charge school fees

Participants were recruited using convenience sampling and with help from educators to try to obtain a representative sample of girls across ages and grades. An educator from each school assisted with the recruitment of learners for the surveys and interviews. Inclusion criteria for the study were learners 16 years and above, educators working in a study school, and mothers who had a daughter enrolled in a study school. The educators who were interviewed were either Life Orientation (LO) educators or educators responsible for the distribution of sanitary products and toiletries, and/or assisting pregnant learners at the respective schools. The mothers who were interviewed had a daughter in the school and either participated in the School Governing Body or worked at the school in some capacity. For the qualititative component, recruitment was halted once data saturation was reached for each group.

### Qualitative procedures and analysis

A female member of the research team conducted female learner FGDs and interviews and a male interviewer conducted the male learner FGDs. Interviews and FGDs were conducted during school hours, in a separate space away from the main school programme. Sessions were audiotaped with permission from participants. The duration for female learner semi-structured interviews was between 20 and 30 min, interviews with educators and mothers between 30 and 45 min, FGDs between 30 and 65 min. In the focus group sessions, participants responded in an anonymous capacity (their names were not recorded). One-to-one interview data was treated confidentially and any names mentioned were anonymized in the transcripts.

Interviews and focus groups were conducted in Sesotho which is the most widely spoken language in Sedibeng and data were transcribed and translated into English. Interviews were reviewed and coded for emergent themes using thematic analysis (Guest et al. 2012). Themes were analysed under two overarching domains of interest that had been determined apriori: menstruation-related needs and challenges in the school setting and impact of challenges on school participation. Emergent themes were identified in each domain through an iterative process including coding, memo writing, and conceptual mapping [23, 24]. Themes were also referenced against existing literature as a means of validation. In the finalisation of analytical themes, discrepancies between codes (across school categories) were discussed and resolved through the method of constant comparison of responses. Meta-themes were derived through triangulation of the qualitative data with the quantitative data. This involved listing the findings from each component of the study and considering convergence, complementarity or discrepancies. An active process of seeking out negative cases or disagreements between the two data was also carried out [25].

### Quantitative procedures and analysis

The quantitative data collection occurred in parallel to the qualitative component. The survey team consisted of a bilingual male and female research assistant. Each female learner independently completed a paper based, self-administered structured questionnaire consisting of 23 items in a group setting during school hours. Fieldworkers gave brief instructions and read out an informed consent form, which learners completed before undertaking the survey or opting out of the study. No learners refused to participate in the study. The questionnaire took 10–15 min to complete.

The survey included questions about age, schooling and race, along with 19 items about MHM. The primary outcome of interest was access to products. This was measured using the question: “In the last three months, did you have enough disposable pads/tampons to last for your whole menstrual period?”. Other items about MHM included age at menarche; acceptability and use of different types of sanitary products; disposal of products (at school and at home); and access, affordability and availability of products. The survey included several questions about absenteeism and menstrual challenges at the most recent period, and about absenteeism and menstrual challenges relating to a period in the last 3 months.

Twenty-eight learners did not meet the age inclusion criteria (the survey was self-administered, and learners who stated an age lower than the minimum age for inclusion in the study of 16 were excluded), and a further five learners were excluded because they did not provide information about their age. Four hundred and seventy-two (472) learners were included in the final quantitative analysis. Descriptive statistics were used to analyse the demographic profile of the learners, and their experiences relating to menstrual health management and sanitary products. Total numbers of learners in the sample along with population proportion estimates and confidence intervals were calculated for each item in the questionnaire, both as aggregates and broken down by school and school quintile to develop an overview of the data that were collected based on a total sample of 472 female learners - the distribution of participants across grades is shown in Table 3. The key findings from this analysis are used to support the findings from the qualitative analysis and presented as part of the key findings in the results section below.

**Table 3:** Distribution of participants across grades

Grade	% (n)
**9**	1.7% (8)
**10**	43.2% (204)
**11**	33.5% (158)
**12**	21.4% (101)

Information about grade was missing from one participant

Few participants in grade 9 were recruited because most learners in grade 9 are not yet 16 years old

A bivariate statistical analyses was conducted to understand the relationship between the primary outcome – access to products – and the key factors of interest, including school quintile, the main product used, the main way of accessing products, affordability, school attendance and the primary challenges faced in relation to MHM based on a total of 447 female learners who answered the question relating to access to products. Pearson’s chi squared tests were used to test for significance, and 95% was used as a threshold for significance. All analyses were conducted in STATA 15. Participants with missing data for access to sanitary products (1.48%) were excluded from the analysis. The reporting of the results followed the STROBE cross sectional reporting guidelines [26].

## Results

### Participant characteristics

All learners who participated in the survey were female (*N* = 472) and were between the ages of 16–22 years, with an average age of 17.5 years. Ninety-five percent (*n* = 444) of the survey participants were black female learners. Sixty-one percent (*n* = 285) of all the learners had ever repeated a grade. The average age of menarche (as self-reported) amongst female learners in our study was 13.36 years, with learners experiencing their periods for an average of 4.07 years. Demographic characteristics for the quantitative survey sample are provided in Table 4.

**Table 4:** Demographic characteristics of participants and key results from descriptive analysis

**Age**		
Mean	17.5 years	SD = 1.3 years
Median	17 years	IQR = 16–18 years
Range	16–22 years	
**Age at menarche**		
Mean	13.4 years	SD = 1.5 years
Median	13 years	IQR = 12–14 years
Range	10–18 years	
	**% (n)**	**95% CI**
**Ever repeated a grade**		
Yes	61.0% (285)	56.6–65.5
No	38.8% (181)	34.3–43.2
Unsure	0.2% (1)	0–0.6
**Race**		
Black	94.0% (444)	91.9–96.2
Coloured	4.7% (22)	2.8–6.6
White	0.4% (2)	0–1.0
Indian	0.2% (1)	0–0.6
Unsure	0.6% (3)	0–1.4
**Sanitary product used most often**		
Disposable sanitary pads	86.0% (406)	82.9–89.2
Panty liners	6.6% (31)	4.3–8.8
Washable sanitary pads	3.2% (15)	1.6–4.8
Tampons	2.3% (11)	1.0–3.7
Other products^a^	0.8% (4)	0–1.7
Menstrual cup	0.2% (1)	0–0.6
Unsure	0.8% (4)	0–1.7
**Sources of sanitary products**^b^		
From family	82.6% (390)	79.2–86.1
From school	33.7% (159)	29.4–38.0
Buy for themselves	20.8% (98)	17.1–24.4
From their boyfriend	3.4% (16)	1.8–5.0
**Method of product disposal while at school**^b^		
Waste bin	67.5% (318)	63.3–71.8
Wait to dispose at home	41.4% (195)	36.9–45.9
Flush down the toilet	10.4% (49)	7.6–13.2
Burn	6.8% (32)	4.5–9.1
Pit latrine	1.7% (8)	0.5–2.9
Do not dispose (washable)	1.5% (7)	0.4–2.6
Bury	1.5% (7)	0.4–2.6

*SD* standard deviation, *IQR* interquartile range, *CI* confidence interval

^a^Including cloth/rags; toilet paper; newspaper and sponges

^b^Percentages do not sum to 100 because participants could choose more than one option

Three major themes with accompanying sub themes were identified in the final analysis; 1) Differential access to and inequitable distribution of modern sanitary products (subthemes: product preferences and acceptability, differential access to modern sanitary products; unintended consequences of an inconsistently applied school-distribution programme); 2) The relationship between menstrual-related challenges, the school environment and schooling outcomes (subthemes: physical discomfort, teasing, and feeling distracted in class, unhygienic sanitation facilities and inadequate rest areas, school absenteeism) and 3) Unmet Sexual and Reproductive Health and Rights Needs of Learners (subthemes: Menarche preparedness and other SRH information needs, gendered and intergenerational barriers to delivery of comprehensive sexuality education).

### Differential access to and inequitable distribution of modern sanitary products

We report the results from a bivariate analysis of the quantitative component (Table 5) together with the relevant qualitative findings under the theme of access to products.

**Table 5:** Bivariate analysis of factors associated with differential access to products in the past 3 months

		Enough products to last for every period in the past 3 months			
		Yes % (n) 84.79% (379)	No % (n) 15.21% (68)	Total % (n) 100% (447)	Pearson’s chi^**2**^***P***-value
**School Quintile**^**a**^	Quintile 1	86.4% (38)	13.6% (6)	100.0% (44)	*P* = 0.882
	Quintile 2	81.4% (35)	18.6% (8)	100.0% (43)	
	Quintile 3	84.4% (189)	15.6% (35)	100.0% (224)	
	Quintile 4	86.0% (117)	14.0% (19)	100.0% (136)	
**Sanitary product used most often**^**b**^	Disposable pads	88.5% (332)	80.9% (55)	87.4% (387)	*P* = 0.237
	Panty liners	6.1% (23)	10.3% (7)	6.8% (30)	
	Washable pads	2.4% (9)	5.9% (4)	2.9% (13)	
	Other	2.9% (11)	2.9% (2)	2.9% (13)	
	Total	100.0% (375)	100.0% (68)	100.0% (443)	
**Sources of sanitary products c**	Family	86.3% (327)	66.2% (45)	83.2% (372)	*P* = 0.000
	School	31.9% (121)	44.1% (30)	33.8% (151)	*P* = 0.050
	Buy themselves	19.5% (74)	22.1% (15)	19.9% (89)	*P* = 0.630
	Boyfriend	3.2% (12)	4.4% (3)	3.4% (15)	*P* = 0.599
**Household ability to afford products when needed**^**b**^	Always	74.2% (279)	25.0% (17)	66.7% (296)	*P* < 0.001
	Sometimes	24.7% (93)	73.5% (50)	32.2% (143)	
	Never	1.1% (4)	1.5% (1)	1.1% (5)	
	Total	100.0% (376)	100.0% (68)	100.0% (444)	
**Ever missed school due to period**^**b**^	Yes	22.5% (85)	46.3% (31)	26.1% (116)	P < 0.001
	No	77.5% (293)	53.7% (36)	73.9% (329)	
	Total	100.0% (378)	100.0% (67)	100.0% (445)	
**Average number of days absent last term**	Number of days	1.9	1.8	1.9	*P* = 0.743
	95% Confidence Interval	(1.7–2.1)	(1.4–2.3)	(1.7–2.1)	
**Main reason for missing school because of period in the last term**^**b**^	Pain	77.4% (65)	51.6% (16)	70.4% (81)	*P* = 0.008
	Not enough products	7.1% (6)	32.3% (10)	13.9% (16)	
	Tired and uncomfortable	6.0% (5)	3.2% (1)	5.2% (6)	
	Unclean toilets	4.8% (4)	3.2% (1)	4.4% (5)	
	Other	4.8% (4)	9.7% (3)	6.1% (7)	
	Total	100.0%)	100.0% (31)	100.0% (115)	
**Most common menstrual-related challenges in last 3 months**^**b**^ **c**	Pain and discomfort	59.2% (222)	44.8% (30)	57.0% (252)	*P* = 0.028
	Distracted during class	20.8% (78)	25.4% (17)	21.5% (95)	*P* = 0.401
	None	20.6% (77)	14.9% (10)	19.7% (87)	*P* = 0.283
	Unable to manage flow	19.2% (72)	35.8% (24)	21.7% (96)	*P* = 0.002
	Didn’t go out with friends	18.9% (71)	10.5% (7)	17.7% (78)	*P* = 0.093
	Could not afford products	5.1% (19)	28.3% (19)	8.6% (38)	*P* < 0.001

*P*-Values from Pearson’s Chi^2^ tests show statistical significance of differences between groups

^**a**^Percentages shown are calculated as row percentages i.e. as a percentage of the total number of participants in each row of data

^**b**^Percentages shown are calculated as column percentages i.e. as a percentage of the total number of participants who 1) had enough products to last for every period in the past 3 months; 2) did not have enough products to last for every period in the past 3 months; and 3) on all participants who answered the question about having enough products

c Participants could choose more than one option. Percentages are calculated on the whole sample. Therefore, percentages should not add to 100%. Pearson’s chi^2^ *p*-values are thus calculated separately for each binary (yes/no) variable listed

#### Product preferences and acceptability

Amongst all the survey participants (*N* = 472), 86.0% of female learners reported preferring or only ever using disposable sanitary pads. Very few learners reported using tampons (2.3%), with the qualitative interviews suggesting discomfort, fear over use, and cultural resistance to use of tampons amongst girls as the main reasons for lack of uptake.

**..** *parents know about sanitary pads only. We don’t know about tampons or we are scared of them, I don’t know. There’s also a myth about a child losing their virginity when using tampons. So parents provide materials which they prefer based on what has or is currently working for them.* (Educator, School D)

Only one learner reported using a menstrual cup. Preference for sanitary products was also influenced by knowledge of product range and the person who purchased them.

*I use pads because my mother only buys me pads.* (Female Learner, School G, PID 19).

#### Differential access to modern sanitary products

Eighty-five percent of female learners included in the bivariate analysis reported having enough sanitary products to last their every period in the past 3 months. One in seven female learners reported not having enough sanitary products for every period in the last 3 months and this was reflected across the school quintiles suggesting widespread differential access. Overall, two thirds of female learners (66.7%) indicated that their family could always afford to buy sanitary materials, just under one third could sometimes (32.2%), and five learners (1.1%) reported that their family could never afford products.

Girls obtained sanitary products from a number of different sources but most commonly reported obtaining products from family members (82.6%). Nearly one fifth of learners also reported sometimes purchasing products themselves (20.7%) and a minority sometimes obtained products through their boyfriends (3.4%). Schools emerged as an important site for accessing products, with one third of female learners (33.7%) reporting receiving products from school – either through a public sector school distribution programme or a non-governmental organisation initiative. The qualitative interviews repeatedly highlighted the supportive nature and value of these school-based initiatives.

*I buy the pads for my child but on a bad month I rest easy as the school provides them with a toiletry pack* (Mother, School J)

Overall, the data pointed to the reality that differential access to sanitary products did negatively impact learners on multiple levels – and particularly so for learners living in financially constrained circumstances.

*Most learners are shy. They tend to feel embarrassed around other learners. As you know these learners are from different backgrounds. Some can afford disposable pads whereas others cannot. Some of these dynamics are responsible for some learners teasing others about how they smell and so forth.* (Educator, School B)

Overall, 96 learners reported being unable to manage their flow (21.7%). However, managing menstrual flow was found to be a significantly greater challenge (Pearson's chi-square *p* =0.002) for those learners who reported having insufficient products over the past 3 months (35.8%), compared to those who had sufficient products (19.2%)

#### Unintended consequences of an inconsistently applied school-distribution programme

Although most of the schools included in the study sample had reportedly received disposable pads at some point, not all learners at the schools had been reached and sustainability of supply emerged as an issue, with many schools and learners reporting that the pads had not been distributed for up to 3 months prior to the study. In some schools, limited supplies resulted in inequitable distribution of pads amongst learners.

*You see Mam these teachers don’t give us pads, you see this teacher who is passing by? She is the one who locks the office and only give pads to her favourites. Some of us cannot afford pads but she says I can afford just because am wearing Chino pants* (good quality trousers) *to school.* *What if my uncle bought them for me? How does she know?* (Female Learner, School H, PID 23)

In schools with limited supplies or lacking in guiding policy on the issue, learners were required to specifically request pads from a designated educator which was widely viewed as embarrassing for the learner concerned, who either sent a friend on their behalf or avoided the school supply and borrowed a pad from a friend.

*..sometimes when some of the girls don’t have pads, they are afraid to ask to such an extent that they send someone else to ask on their behalf... It’s like an embarrassing and shameful request to make.* (Educator, School J)

The circumstance of being too embarrassed to request pads and not being confident to deal with their menses at school was reportedly worse for younger learners, than with older learners.

*[When they are menstruating] they are not as involved as other days Especially in the lower grades; the seniors are just fine from grades 10 until 12, but in grades 9 and 8, you see elements of them being embarrassed. I think it is because they are experiencing a process that is fairly new to them.* (Educator, School C)

In a number of schools, educators reported that they contributed personal funds so the school could purchase pads for emergency situations or for learners from financially constrained backgrounds. However, this was solely at the discretion of the individual educators at the different schools, and was insufficient to meet demand as well as being unsustainable.

### The relationship between menstrual-related challenges, the school environment and schooling outcomes

There was a complex interaction between the menstrual challenges experienced by female learners, often amplified or compounded by factors in the school environment, and schooling participation and attendance.

#### Physical discomfort, teasing, and feeling distracted in class

While access to products was a critically important challenge facing some learners, the most widely reported challenge facing all girls during their menses was dysmenorrhea. More than half of the learners (*n* = 252, 57.0%) listed pain as one of the main challenges they faced in managing their menses in the past 3 months. This was the most frequently reported challenge, both amongst girls who reported having enough products to last their whole period (*n* = 222, 59.2%) and among those who did not (*n* = 30, 44.8%). Feeling distracted and inability to concentrate in class was another reported challenge amongst learners (*n* = 95, 21.5%), all of which were echoed amongst mothers and educators, who also indicated limited ability to assist.

*Concentration is low to slow especially when they are at the beginning of their period. Because of menstrual pains, you’d find many of them sleeping on their arms and when you ask whether they are sick or not, the response is always menstrual pains. But we are not permitted to give such a learner medication. Half the time we give them warm water to drink*. (Educator, School I)

Feelings of anxiety around having soiled oneself negatively affected classroom participation, with female learners reporting reticence to go to the front of the class to write on the chalkboard and needing to be excused more frequently from class to go to the bathroom.

*When they are at school they frequently have to go to the toilet which disturbs lessons so they manage their menstruation by wearing bigger pads which are meant for bigger flow.* (Mother, School I)

Educators identified that younger learners who were still learning to manage their menses appeared to struggle more with these challenges than their older counterparts.

*… in FET (senior phase) classes dealing with menstruation is not such a big deal. The learners are confident to a point that they would say “ma’am may I please be excused I just need to go change [my pad].”* (Educator, School C)

While frequent requests to go to the bathroom may be disruptive to the class lesson, refusal of some educators to allow female learners to go to the bathroom until the has class ended compounded feelings of anxiety and distraction.

*Some teachers do not allow you to go to the toilet, you must wait until break time. By the time its break time you have soiled on yourself, now the learners laugh at you.* (Female Learner FGD, School G)

The teasing of menstruating learners was raised by all study participants, including male learners. However, it appeared that this teasing was not only limited to menstruating learners but sexually active female learners more broadly. One of the research assistants directly observed a situation where some male learners addressed female learners as “*difebe tsao preventa*”(loose girls who use prevention) in front of an educator. According to the research assistant, the educator laughed at the comment and walked away without intervening. Male learners participating in the focus group discussions acknowledged that teasing did occur and that some educators did take action to put a stop to it.

Respondent: [Teasing] happens quite a lot*.*

Interviewer: What do they say?

Respondent: Caution wet floor ahead*.*

*Group laughter*

Interviewer: How do teachers react if they see boys teasing girls?

Respondent: Some teachers reprimand you on the spot. To an extent which your flaw is made public to compensate for your teasing of the girl*.*

(Male Learner FGD, School I)

#### Unhygienic sanitation facilities and inadequate rest areas

Menstrual hygiene needs include clean, functional and private toilet facilities, appropriate method of waste disposal of used sanitary products and availability of soap and water within the facility. Two primary disposal methods for used sanitary products were reported by the female learners who participated in the survey: soiled sanitary products were either thrown in the bin or bucket provided (67.5%) or stored in their bags to be thrown away at home (41.4%). Inadequate sanitation and waste disposal facilities appeared to be the key reason behind many learners keeping their soiled pads in their bags to dispose of later at home.

Respondent: I always have an extra pad in my bag and when I change my pad, I roll it up and put it in my bag and I throw it when I get home*.*

Interviewer: Is there a specific reason why you only throw your pads at home?

Respondent: There are no bins at school and I don’t like it when other girls throw pads all over the floor so I just put it in my bag*.*

(Female Learner, School G, Interview 21)

Less common disposal methods included throwing sanitary pads in the toilet (or pit latrine), burning or burying them. Disposal methods did not widely differ between schools.

The lack of cleanliness and poor condition of learner toilets was consistently highlighted by all categories of study participants, including an independent assessment conducted by the research team via a structured checklist. The assessment found that many of the school facilities had leaking toilets or basins, some had muddy water over the floor; in addition there were used sanitary pads, tissues, chip packets and sweet wrappers on the floor. The toilets themselves were reported to have a strong urine smell. Overall, two schools had female toilets that were reported as clean, 6 schools as having somewhat clean toilets and one school not at all. The latter school also had partially functioning learner toilets. One of the schools had the windows sealed shut by paint which meant no ventilation was possible. No soap was reported in any of the learner toilets in all of the schools. Only two schools had bins for disposal of soiled sanitary materials in the toilet stalls, the balance of schools had no bins at all for soiled sanitary materials in their learner toilets. The state of the available ablutions was repeatedly raised by female learners for whom “home” was synonymous with clean facilities and school with dirty ones.

*I cannot use the toilets because there is no water which makes me not to focus because all I am thinking about is the clean toilet at home that I can use to change my pads. You have to wear this big night pad so that it can last you until after school and then next thing you are smelling … I cannot focus.* (Female Learner, School D, Interview PID 11)

The issue of inadequate or unclean facilities, however, is complex because it appeared that the toilets were cleaned and resupplied in the mornings but that soap and toilet paper quickly ran out, as well as the deteriorating nature of the hygiene conditions of the toilets. And even when bins were provided they were not necessarily made use of. Lastly, the lack of a sick bay or appropriate rest area in some of the schools was raised by all groups of participants as a gap in support that the school could offer to menstruating learners.

*Privacy [is needed] which can be provisioned by making a designated space for menstruating girls to come recuperate and then return to class. However, each time they are sent to the kitchen for warm water [to drink] and made to sit at reception; and that place is cold.* (Mother, School A)

Interestingly, male learners echoed that menstruating girls required a rest area and highlighted the gendered sensitivities impacting on comfort levels of menstruating girls.

*They need their own designated sick-room because right now the staff room is being used as a sick-room. Male teachers are present there and this may hamper them from feeling comfortable thus disclosing to the female what they really need.* (Male Learner FGD, School H)

#### School absenteeism

In the bivariate analysis, one quarter of respondents (26.1%) reported that they had ever missed school due to their period, with those who didn’t have enough products over the last 3 months more likely to miss school than those who reported sufficient products (46.3% vs 22.5% respectively, *p* < 0.001). However, there was no statistically significant difference between the groups in number of days missed – overall learners on average missed 1.9 days school in the last term (1.69–2.08, 95% CI) – suggesting that girls experience multiple challenges during menstruation, any combination of which may impact school attendance. Of all the girls who reported missing school, pain was most commonly reported as the main reason for missing school (*n* = 81, 70.4%). Teasing also had a role to play. When asked why a learner may miss school when she has her period, one learner replied:

*There is also no protection from teasing, teachers just walk away or ignore it.*

(Female Learner FGD, School G)

Missing school on account of menstruation was not viewed as an option by educators and mothers who were qualitatively interviewed. One mother indicated that she allowed her daughter to stay home for one day if she experienced particularly painful menstrual cramps. Female learners also made no argument for missing school, with some preferring to be at school instead of being assigned household chores should they remain at home. It appeared more common for learners to come to school initially and then asked to be excused from school early on account of pain or accidentally soiling their clothes.

### Unmet sexual and reproductive health and rights needs of learners

Levels of knowledge around menstruation were widely variable and learners expressed a number of unmet SRH information and support needs.

#### Menarche preparedness and other SRH information needs

While female learners in the study demonstrated sound understanding of personal hygiene needs during their menses and understood that they could now conceive a child, very few could correctly explain or understood the menstruation process, with patchy and often incorrect knowledge being offered. For some, menstruation was the release of “dirty blood” or “blood coming from their ovaries”. Others had no explanation or understanding as to why menstruation occurred.

*No, I don’t know [why girls and women menstruate]. I just know that dirty blood must come out and you must count 28 days before it comes out again but I do not know what happens inside*. (Female learner, School D, PID 12)

Interestingly, some male learners in the FGDs displayed greater accuracy in understanding the reason for menstruation than some of the female learners. In most of the male learner focus groups, the male learners believed it was important for boys to understand and know about the topic of menstruation, either because they thought they may have daughters one day and needed to be in a position to provide appropriate advice or through their reported need to understand their girlfriend’s experience in terms of how they felt and what they went through each month.

Levels of knowledge around the menstrual process positively or negatively shaped girls’ experiences of menarche. In the individual interviews, female learners reported variable reactions to menarche, with some learners recalling feelings of excitement and others recounting menarche as a distressing experience because they had no understanding as to what was happening to their bodies. The majority of learners reported being inadequately prepared for menarche.

*I think I was 13. It happened at night when I started feeling sick. The next morning it got worse, then I saw blood on my panties and I started panicking thinking which guy did I sleep with because my mom had told me that if I have sex with a boy I will bleed. I washed my underwear and I hid it under the bed.* (Female Learner, School C, PID 9)

On menarche, most girls reported approaching or being approached by a female family member or educator who assisted with helping them make sense of the experience. Educators played a key role in normalizing the experience for some students, some of whom chose to approach an educator rather than a parent. Information shared by female relatives tended to focus on appropriate behavioural conduct and to “stay away from boys” rather than on developmental or biological processes.

Some learners indicated that they had learned about menstruation already in class but there was clear variability in scope, timing and frequency of these lessons with both learners and educators calling for greater depth and application over the issue. All learners highlighted significant gaps in the quality and consistency of the delivery of sexuality education (which is included in Life Orientation lessons as part of the South African school curriculum). The need to have an iterative conversation about menstruation throughout the Grades was also a clear message from both learner and educators.

*We need to figure out how we can talk more about it in the curriculum, but like I said in the current textbooks it’s like a paragraph... Teachers need to explore more than just that paragraph. Teach testable things to teach but also equip learners with the necessary life skills.* (Educator, School J)

The pervasiveness of irregular menstrual patterns (some of which may have been related to contraceptive side effects) was one key information gap highlighted by the types of questions raised by some of the female learners, who sought to understand why their menstrual cycles differed from the established 28 day “norm”.

**Respondent 1:** What happens when you start your periods and then they stop? I went on my periods in 2013 and in 2014 they stopped. Won’t that give me problems?

**Interviewer:** First how old were you?

**Respondent 1:** 15 years and they’ve stopped ever since. I went to the clinic and got a referral letter to go see a doctor, but he saw nothing wrong with my situation*.*

**Respondent 2:** I skip a month when I have my periods*.*

**Respondent 3:** Some go for one day*.*

**Respondent 4:** I’d go on for an entire month*.*

**Interviewer:** You went to the clinic for this?

**Respondent 4:** I did and they gave me pills to drink, but when I didn’t take this pill it started all over again, until I got an implant*.*

(Female Learner FGD, School B)

There were also clear broader unaddressed SRH information needs. Our female research assistant fielded an array of questions from female learners after the interviews formally ended, with advice being sought on a range of issues, including alternative contraceptive methods that didn’t cause the side effects of irregular periods, weight gain and heavy menstrual flow. In one school, some FGD participants had already mothered a child and wanted to understand why they had become pregnant given that they had followed the “6 day rule” (ie. the belief that pregnancy cannot occur if one engages in unprotected sex within 6 days post menstruation). Both during and after interviews, female learners called for more information and for counsellors/experts to come to their schools and engage in talks with them and give them guidance on a range of SRH and relationship issues. They shared difficulties in talking to their parents about boyfriends and sex and being too scared to attend the clinics for advice.

*Mam we need education, these teachers don’t teach us about everything you have been talking about. We need a person who can come to our school at least once a week to educate us and give us support because these teachers sometimes swear at us and say we are using contraceptives. We also need a school nurse, if you go to the clinic for help. Yooh they will swear at you until you leave*. (Female Learner, School G, PID 21)

#### Gendered and intergenerational barriers to delivery of sexuality education

While sexuality education is part of the South African Life Orientation curriculum, gender and social norms emerged as barrier to provision of comprehensive sexuality education. Male educators, in particular, expressed discomfort in teaching on the topic of menstruation.

*As a male teacher I don’t feel comfortable talking about it. Because with us Africans talking about sexual health with young girls can be very awkward.* (Educator, School H)

This discomfort may result in male educators avoiding the topic all together.

*[The male teachers] don’t teach us about menstrual hygiene. They talk about sporting activities, eating healthy and exercising.* (Female Learner FGD, School G)

Male learners also identified educator-related barriers to learning about menstruation, including male educators’ avoidance of the topic or educator’s reluctance to have open discussions, including veiled allusions to the topic which made it difficult to distinguish fact from fiction or one-sided perspectives based on educator’s personal experience.

*Female teachers also share this information from their own understanding and this does not mean we get the fuller picture. We are told these things from their personal experience.* (Male Learner FGD, School D)

Not only does the male educator’s discomfort result in a missed opportunity to educate male learners on reproductive health but male educators’ own lack of knowledge over the menstrual process also has the potential to result in lack of support to menstruating learners.

*Normally, as male educators we don’t know how these learners feel when they are menstruating. If it could be explained to us as to how the cycle affects them in terms of symptoms pre and post their period. I have come to realize that the learners tend to become moody and then as male educators we would react aggressively which may end up making the child feel uncomfortable. This in turn creates an unconducive atmosphere in the classroom. Maybe, if we knew all of this or even half, our reactions as male teachers would be better than what I’ve just explained.* (Educator, School B)

Lastly, in the home environment, while some male learners reported that their mothers had provided them with information on menstruation, this was not commonly a topic of conversation between many parents and children, reinforcing the importance of accurate and quality sexuality education within schools.

## Discussion

Our findings illustrate the complex relationship between the menstrual-related challenges experienced by female learners in our study, mediated by gendered and structural dynamics within and outside the school environment, and the concomitant impacts on their right to dignity, school participation and attendance. Adolescence marks a period of tremendous physical, psychological and cognitive change, with adolescent development trajectories and their health outcomes profoundly shaped by their structural and social environments – including the school environment in which learners spend a significant portion of their day. Some adolescents will experience greater health challenges than others through being differentially situated in terms of risk for poor health, with strong links between socioeconomic status and health behaviour and outcomes [1]. In this context, a well-designed, consistent and sustainable formal school-based sanitary product distribution programme is crucial, especially in schools that serve poor communities. However, this programme must be situated within an iterative, high quality and consistently applied SRH educational programme that addresses the myriad of reproductive health challenges that girls face, including and following menarche. Further, schools need to be supported to increase the availability and quality of sanitation facilities to ensure girls are enabled to properly manage their menses without the risks relating to poor hygiene, social ridicule or negative environmental impact. Creating a policy environment that takes into account the systemic and structural complexities that impact girls’ and young women’s right to education (including comprehensive sexuality education) and right to dignity will establish an important foundation for implementation of school-based reproductive health programmes in the future.

Age at menarche is a key clinical indicator of a girl’s physical maturation and nutritional status as well as an important indicator of socioeconomic differentials and reproductive potential [27]. However, the age of menarche is not routinely captured in population level surveys and is a data gap, particularly in sub-Saharan Africa, which means that comparisons can only be derived from other studies [27]. In a study conducted in Uganda, the average age of menarche was 14 years [28]. The age of menarche in our study population compared to a number of other South African studies, including an earlier study which reported a mean age of 13.2 years in the same population group [29]. In another South African study conducted in 2000 amongst 15–19 year old adolescents, the age of menarche was 13.6 years [30] and in a 2013 South African study conducted amongst 18–45 years old, the age of menarche was 14.7 years [31]. Another two other South African studies which report an average age at menarche of 12.5 years [32] and 12.66 years [33] amongst black South African girls. Although this was a small sample size and only conducted in one district of Gauteng, our age of menarche was comparable to other South African studies.

Our study reflected broadly similar findings of recent reviews which have highlighted that girls in low and middle income countries generally have inadequate understanding of menstruation and are not prepared for menarche [5, 14, 34]. These reviews also identify mothers and other female family members as the main sources of menstrual information for young girls but highlight that these adult groups often have gaps in their own knowledge [34]. In our study, female educators emerged as an important resource for female learners in helping them through menarche or providing support at school during menstruation. However, male educators may require better support in understanding and teaching on MHM.

There were clear information needs amongst both male and female learners participating in our study. Female learners may require greater support in understanding different menstrual patterns and flows and education on a myriad of other SRH needs and concerns, including effective pregnancy prevention technologies that don’t negatively impact their menses. While the relationship between teenage pregnancy and school dropout is also complex, teenage pregnancy is a key factor underlying school drop-out amongst female learners in the region [35], particularly in country contexts where educational policy does not permit learners to return to school after giving birth. Interestingly, there have been a small number of school-based interventions to determine the impact of provision of modern sanitary materials on schooling outcomes [6, 36–39]. In the course of these studies, there is some indication that puberty education interventions may have as good or greater effect on schooling attendance as only provision of sanitary products [37, 38].

Reaching all learners with age appropriate sexuality education, however, is a challenge in the South African school setting. Our findings that many female learners had repeated a grade are consistent with a recent analysis of the South African Department of Education data which found that as male and female learners progress in secondary school, there are increases in levels of grade repetition, particularly from Grades 8–11 for all learners [40]. While this may be indication that learners are not sufficiently prepared in the foundational school years, in the context of our findings flag some of the difficulties in ensuring adequate comprehensive sexuality education (CSE) and SRH support to a wide age range of learners studying in the same grade (with up to a six year age gap between the youngest and oldest learner in a given grade),. This points to a likely mismatch in age appropriateness and level of information delivered in each grade and may require CSE targeted at age of learners rather than grade.

Our findings regarding the preference and acceptability of disposable sanitary pads offer guidance to public sector efforts to provide sanitary products to female learners. However, the sustainability of the provision of pads (rather than alternative modern sanitary products) in terms of burden on waste disposal systems and environmental impact will need to be addressed. There have been promising efforts in other countries in the region to introduce menstrual cups in schools which are environmentally sustainable, cost effective, safe and have shown the best health outcomes [6, 39, 41]. The availability of menstrual cups is relatively new in South Africa and has largely had exposure amongst women (18–24 years). However, there may be several cultural barriers to use in younger women under 18 years and shifting to more environmentally sustainable products amongst younger cohorts may take some time. However, the foundations for the shift should begin now, at policy and curriculum level and through social marketing strategies, supported by research to better understand what some of the challenges and obstacles to uptake and use may look like amongst female learners in the school context.

### Study limitations

This was an exploratory study conducted in one district in Gauteng which means the findings are not necessarily generalizable to the entire population of South African female learners. Further, the quantitative analysis in this paper focuses on bivariate correlations and thus the relationships found between the key covariates and primary outcome variable may not represent the true net effects. Future research could be designed to capture a more diverse population and examine in more detail other factors that could further explain our results. However, there was high level of convergence between the qualitative and quantitative findings. Only learners 16 years and older were included in this study. The findings, however, highlighted that understanding the experiences of younger girls who have recently experienced menarche is important in that there is indication that they will have particular support needs which may need to be accounted for in policy documents or implementation plans.

## Conclusions

Our findings add to the growing evidence that the provision of sanitary products is only one component of a comprehensive MHM response and that this response needs to be located within a broader SRH framework. Ongoing focus over the link between product access and absenteeism risks excluding consideration of other complex systemic and structural factors which can negatively impact the SRH of learners in the school context, and more broadly. These include the need for increased efforts to provide iterative, high quality and accurate SRH information and support to learners, more work to be done amongst educators in increasing their reproductive health knowledge, including gender sensitization and values clarification, ensuring that school sanitation facilities are hygienic, private and safe, including adequate waste disposal containers and, more broadly, environmentally sustainable waste disposal systems.

## Data Availability

The datasets used and/or analysed during the current study are available from the corresponding author on reasonable request.

## Abbreviations

ESA: Eastern and Southern Africa
FGD: Focus Group Discussions
LO: Life orientation
MHM: Menstrual health management
SRH: Sexual and reproductive health
